# Supplementary material for: Field Trial of an Automated Batch Chlorinator System at Two Shared Shallow Tubewells among Camps for Forcibly Displaced Myanmar Nationals (FDMN) in Cox’s Bazar, Bangladesh
Source: Int J Environ Res Public Health. 2021 Dec 8;18(24):12917. doi: 10.3390/ijerph182412917 (PMC8701840; doi:10.3390/ijerph182412917)
Supplement: Supplementary file 1 [file ijerph-18-12917-s001.zip › ijerph-1362526-supplementary.pdf]

Article

Field Trial of an Automated Batch Chlorinator System at Two Shared Shallow Tubewells among Camps for Forcibly Displaced Myanmar Nationals (FDMN) in Cox's Bazar, Bangladesh

Nuhu Amin [1,2,*], Mahbubur Rahman [1], Mahbub-Ul Alam [1], Abul Kasham Shoab [1], Md. Kawsar Alome [3], Maksudul Amin [3], Tarique Md. Nurul Huda [1] and Leanne Unicomb [1]

Supplementary Materials

Table S1. Free residual chlorine concentration level with different iron concentrations in 4 tubewells, Cox's Bazar-2017 .

Sampling Dates	Tubewell ID	Concentration of iron (mg/L)	~5.25% NaOCL: Water (mL)	Concentration of chlorine used for treatment	Concentration of Free Chlorine (mg/L)
8/28/2018	17*	<0.2	1500:750	3.5%	9.1
8/28/2018	17	<0.2	1500:750		8.6
8/28/2018	17	<0.2	1500:750		9.3
8/28/2018	17	<0.2	1500:750		10.3
	Mean free chlorine concentration (SD)				9.3 (0.7)
8/29/2018	13	6.5	1500:750	3.5%	3.5
8/29/2018	13	6.5	1500:750		0.02
8/31/2018	13	6.5	1500:500	3.9%	0.4
8/31/2018	13	6.5	1500:500		0.4
	Mean free chlorine concentration (SD)				1.11 (1.6)
8/29/2018	2**	7	1000:500	3.5%	0.04
	Mean free chlorine concentration (SD)				0.04
8/31/2018	5	1.5	1500:750	3.5%	4.2
8/31/2018	5	1.5	1500:750		2.6
8/31/2018	5	1.5	1500:750		4.4
8/31/2018	5	1.5	1500:750		3.7
	Mean free chlorine concentration (SD)				3.73 (0.83)

*At the beginning of the field trial, we had installed Zimba at deep tubewell ID-17, but we received complaints from the household users that the handle had become too tight to pump water. Hence, we removed the Zimba from tubewell ID-17. **Tubewell 2 had a very high (7 mg/L) concentration of iron and it was not providing sufficient residual chlorine immediately after treatment. Additionally, the color of the water became red due to a chemical reaction between iron and residual chlorine immediately after mixing resulting in the removal of the Zimba from tubewell ID-2.

Table S2. A comparison table among WHO recommended drinking water quality, pre-treated water quality, and post-treated (zimba) water quality.

Parameters	WHO	Pre-treated source water	Pre-treated stored water in household own vessel	Post-treated (zimba) source water	Post-treated (zimba) stored water in household own vessel	Post-treated (zimba) household stored water in project provided safe storage containers
		Mean	Mean	Mean	Mean	Mean
[1] Iron (mg/L)	0.3-3.0	4.0	2.2	5.8	1.9	2.8
[1] TDS (ppm)	<300	240.3	238.0	215.3	253.1	235.4
[1] Free Chlorine (mg/L)	0.2-2	NA*	0.15	2.1	0.39	1.4
[1] Total Chlorine (mg/L)	0.2-2	NA	0.17	2.21	0.5	1.72
[2] Turbidity (NTU)	≤5	2.55	2.6	2.50	1.8	2.81

NA* = Not applicable for underground source water. [1] WHO (1996): Guidelines for drinking-water quality, 2nd ed. Vol. 2. Health criteria and other supporting information. World Health Organization, Geneva, 1996. [2] WHO (2017): WATER QUALITY AND HEALTH - REVIEW OF TURBIDITY: Information for regulators and water suppliersWorld Health Organization, Geneva, 2017.

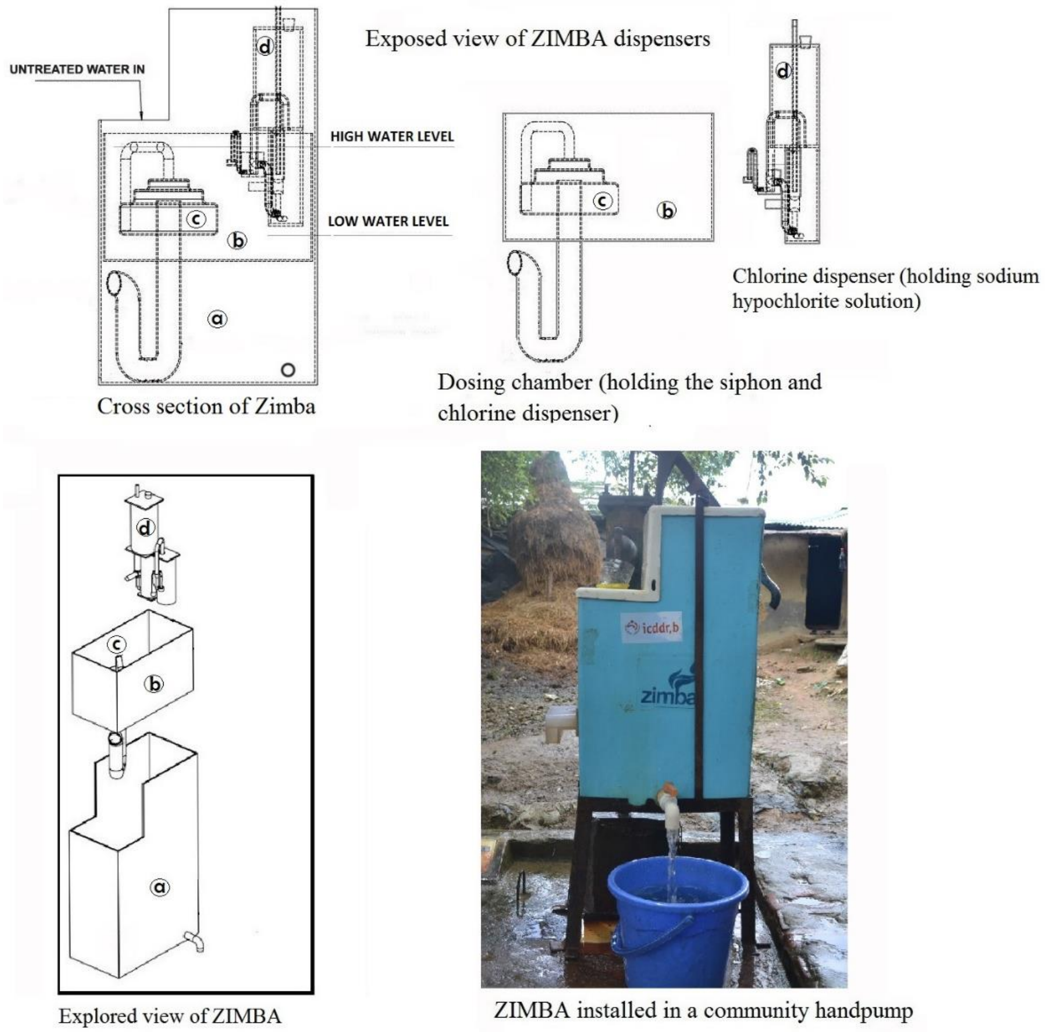

Figure S1. Zimba automated chlorine dispenser. Figure provided by inventor Suprio Das. Figure showing (a) Outer box: upper part of the outer box holds dosing chamber and lower part acts as a secondary tank which water flushes into after chlorination. (b) Dosing chamber: this chamber holds an automatic siphon and the chlorine dispenser. As untreated water from the handpump starts filling up this chamber, 3 mL of sodium hypochlorite solution is ejected from the chlorine dispenser into this water. When the water level reaches the high-water level (10 L) the automatic siphon is triggered and this 10 L of treated water is flushed into the secondary tank. (c) Siphon: Water from the dosing chamber flushes into the secondary tank through the siphon. (d) Chlorine dispenser: This consists of a chlorine reservoir and a combination of interconnected pipes and tubes. Dimensions of the Zimba are 76 cm x 45 cm x 25 cm; the outer casing, dosing chamber, and the siphon are made of fiberglass and the dispensers are made of acrylic.

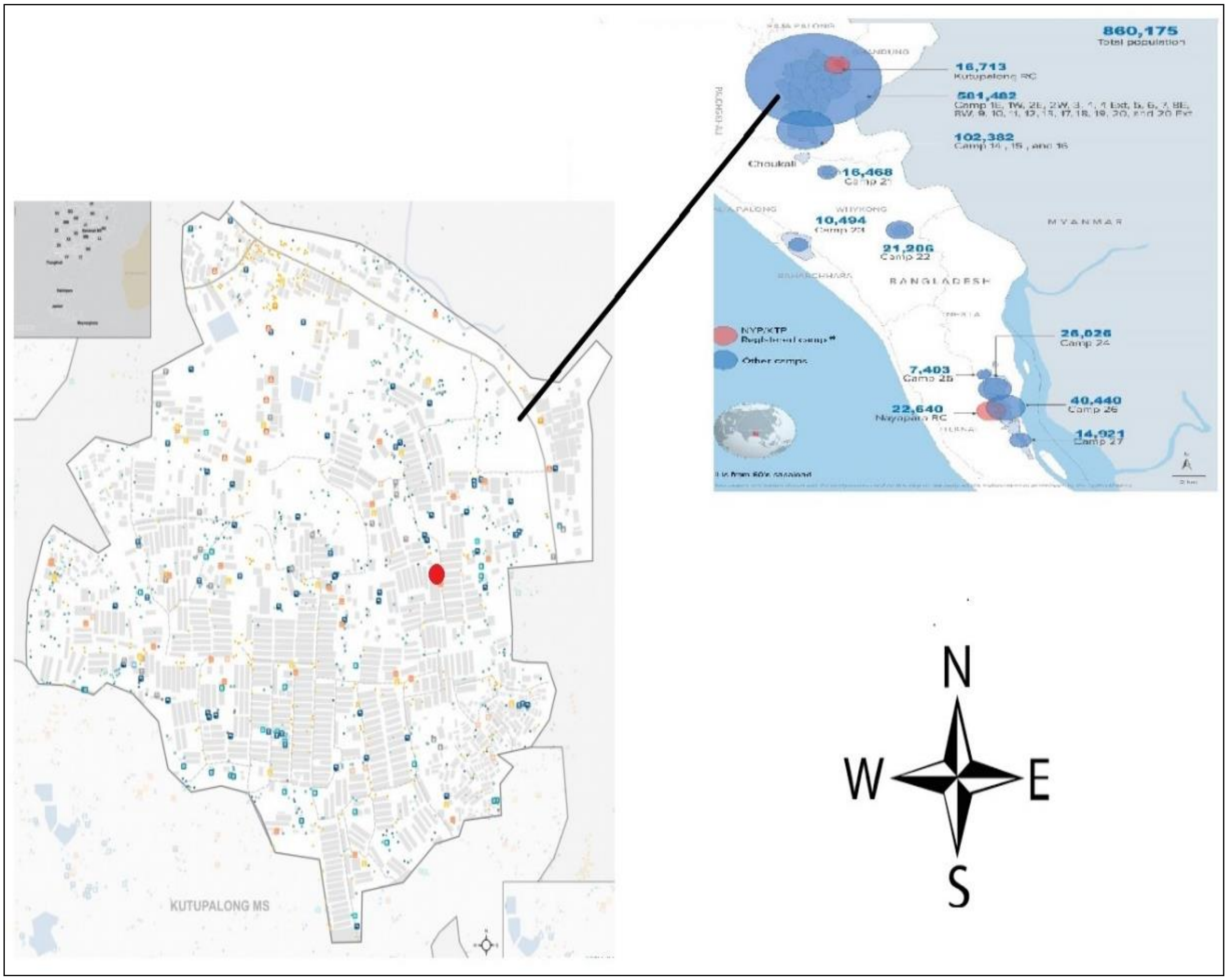

Figure S2. Study site map.

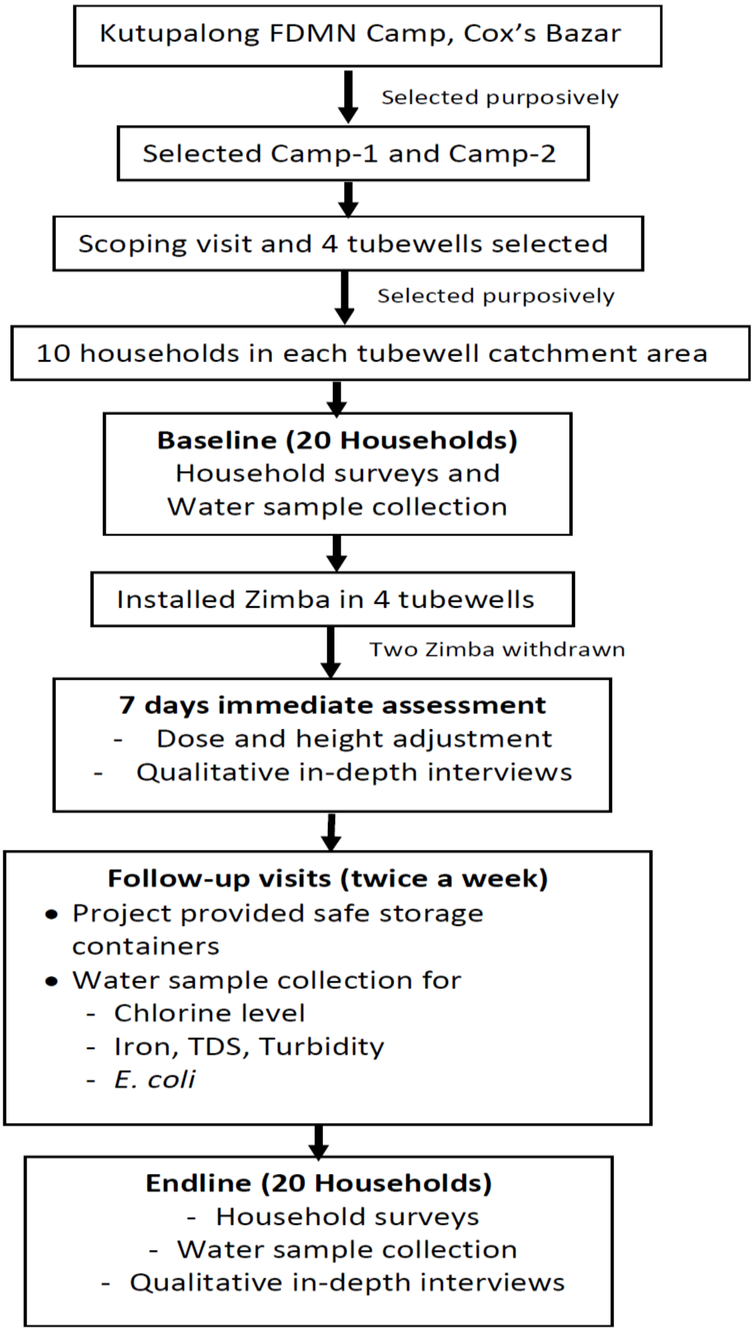

Figure S3. Study flow chart.

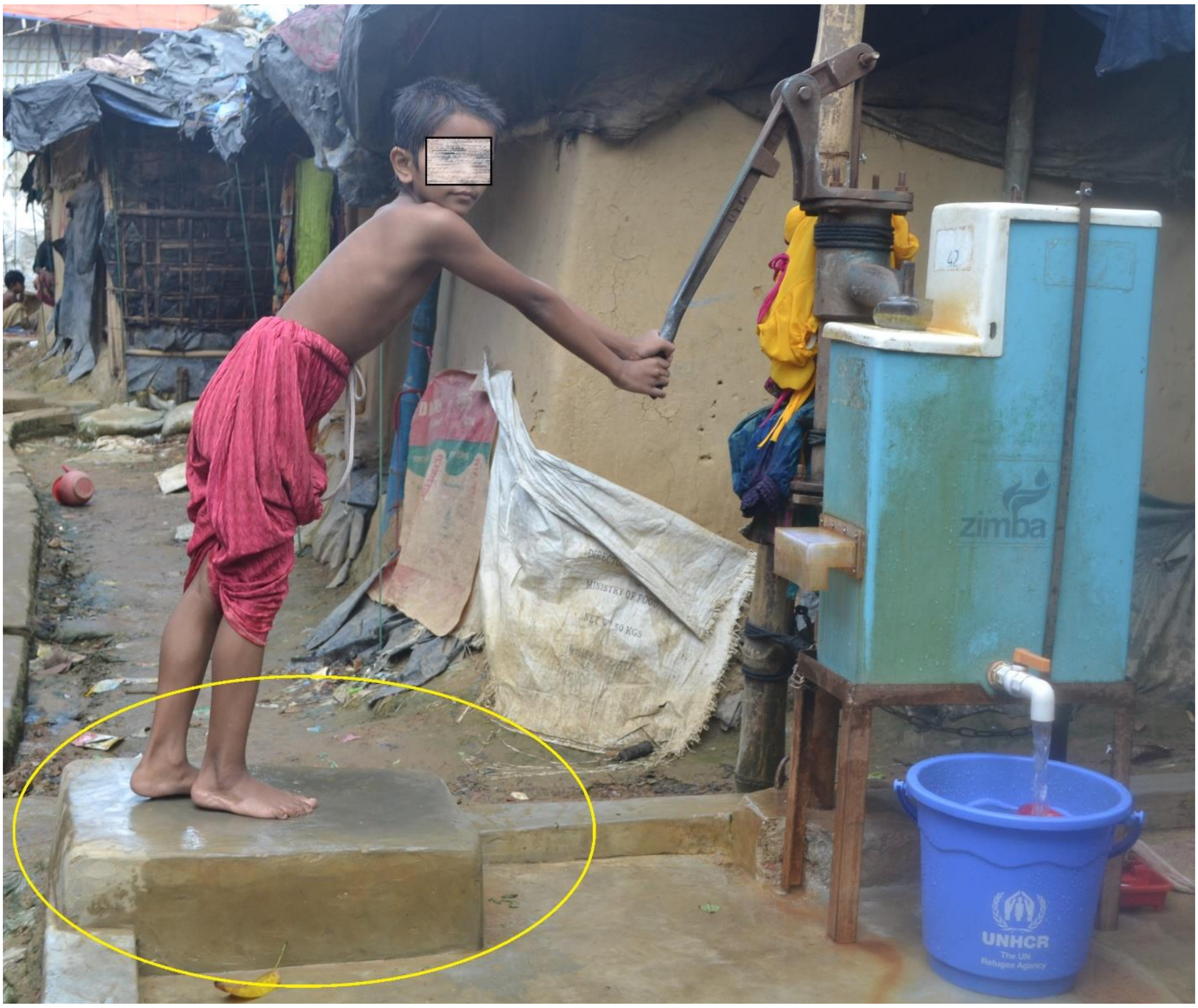

Figure S3. Raised footstep to resolve the increased height of tubewell after Zimba installation, Rohingya camp, Cox's Bazar-2017.
